# Atrophy and Inflammatory Changes in Salivary Glands Induced by Oxidative Stress after Exposure to Drugs and Other Chemical Substances: A Systematic Review

**DOI:** 10.3390/medicina59091692

**Published:** 2023-09-21

**Authors:** Loredana Beatrice Ungureanu, Irina Grădinaru, Cristina Mihaela Ghiciuc, Cornelia Amălinei, Gabriela Luminița Gelețu, Cristina Gabriela Petrovici, Raluca Ștefania Stănescu

**Affiliations:** 1 Morphopathology, Department of Morpho-Functional Sciences I, Faculty of Medicine, Grigore T. Popa University of Medicine and Pharmacy, 16 Universitatii Street, 700115 Iasi, Romania; loredana.ungureanu@umfiasi.ro; 2 Department of Implantology, Removable Prostheses, Dental Prostheses Technology, Faculty of Dental Medicine, Grigore T. Popa University of Medicine and Pharmacy, 16 Universitatii Street, 700115 Iasi, Romania; 3 Pharmacology, Clinical Pharmacology and Algeziology, Department of Morpho-Functional Sciences II, Faculty of Medicine, Grigore T. Popa University of Medicine and Pharmacy, 16 Universitatii Street, 700115 Iasi, Romania; 4 Histology, Department of Morpho-Functional Sciences I, Faculty of Medicine, Grigore T. Popa University of Medicine and Pharmacy, 16 Universitatii Street, 700115 Iasi, Romania; 5 Department of Surgery, Faculty of Dental Medicine, Grigore T. Popa University of Medicine and Pharmacy, 700115 Iasi, Romania; gabriela.geletu@umfiasi.ro; 6 Infectious Disease, Department of Medical II, Faculty of Medicine, Grigore T. Popa University of Medicine and Pharmacy, 16 Universitatii Street, 700115 Iasi, Romania; cristina.petrovici@umfiasi.ro; 7 Biochemistry, Department of Morpho-Functional Sciences II, Faculty of Medicine, Grigore T. Popa University of Medicine and Pharmacy, 16 Universitatii Street, 700115 Iasi, Romania; raluca.stanescu@umfiasi.ro

**Keywords:** oxidative stress, drug-induced lesions, chemically-induced lesions, salivary glands, histopathology, oxidative stress biomarkers

## Abstract

*Background and Objectives:* Oxidative stress is involved in the alterations at the level of salivary glands, being the cause of oral pathologies like xerostomia, periodontitis, gingivitis, leucoplakia, and cancer. It is known that antioxidants can reverse changes induced by drugs or other chemicals in some organs, but the question is whether these substances can reduce or revert the effects of oxidative stress at the salivary gland level. Our aim was to find histopathological data at the level of salivary glands supporting the hypothesis of the reversal of oxidative stress-induced changes after the treatment with substances with antioxidant effect. *Materials and Methods*: A systematic search was conducted in PubMed, Science Direct, and Springer databases, including research articles on oxidative stress histological aspects and oxidative stress biomarkers induced by drugs or other chemicals on salivary glands. *Results*: Out of 1756 articles, 25 articles were selected with data on tissue homogenate used for biochemical analysis of oxidative and antioxidative markers, along with routine hematoxylin eosin (HE) and immunohistochemical analysis used for histopathological and immunohistochemical diagnosis. Drugs (antineoplastic drugs, antibiotics, and analgesics), alcohol, heavy metals, and fluoride can cause oxidative stress, resulting in morphological changes in different tissues, including in salivary glands. There are many antioxidants but only a few were evaluated regarding the effects on salivary glands in animal studies, such as hesperidin and selenium, which can reverse the damage induced by cyclophosphamide; 10-dehydrogingerdione (10-DHGD), a compound extracted from ginger, which has a protective effect against the oxidative stress and apoptosis induced by tramadol; and glycyrrhizic acid, which may repair the injuries incurred after the administration of sodium nitrite. *Conclusions*: Substances such as hesperidin, selenium, 10-dehydrogingerdione, and glycyrrhizic acid are antioxidants with proven restorative effects on salivary glands for the damage induced by oxidative stress after exposure to drugs and other chemical substances; however, demonstrating their similar effects in human salivary glands is challenging.

## 1. Introduction

Oxidative stress, through the imbalance between oxidants and antioxidants in favor of the former ones, is involved in the alterations at the level of salivary glands. Factors from the oral cavity, such as microorganisms, food, alcohol, medication, tobacco, and fluoride [1,2], trigger proinflammatory cytokines, alter salivary flow, and composition [2] and may generate reactive oxygen species (ROS) that induce oxidative stress. Increased ROS production in the oral cavity causes lesions, such as xerostomia, periodontitis, gingivitis, aphthous stomatitis, osteitis, oral leucoplakia, and oral cancer [1,2,3]. Among these, xerostomia is linked to the activity of salivary glands, being one of the most fearful complications since it impairs the quality of life.

Enzymatic and nonenzymatic antioxidative systems, found also in saliva and in salivary glands, are used to counteract the toxic effects of ROS [4,5]. The activities of key enzymatic antioxidants of the antioxidant system, such as superoxide dismutase (SOD), catalase (CAT), and glutathione peroxidase (GPx), are very low in saliva, compared to the same parameters measured in blood [5]. On the other hand, it was reported that the metabolism of parotid glands (PG) is essentially aerobic and is better protected against oxidative stress, while that of submandibular glands (SMG) is mainly anaerobic and therefore need less oxygen [4,6,7,8,9,10].

Exposure to some drugs and environmental chemicals might result in the oxidation of cellular lipids and proteins [2,11,12] and in cell death induced by cell component destruction and DNA damage [12]. Drugs such as antineoplastic drugs (cyclophosphamide and 5-fluorouracil), antibiotics (metronidazole), and analgesic drugs (tramadol) are known to produce lesions at the level of salivary glands. Animal studies provide strong indirect evidence to support the hypothesis that antioxidants can reverse changes induced by drugs or chemicals in salivary glands. However, demonstrating the antioxidant effects of certain substances in human salivary glands remains challenging [13,14]. Most of the studies have used tissular homogenate for biochemical analysis of oxidative and antioxidative markers, along with routine hematoxylin eosin (HE) and immunohistochemical analysis for histopathological and immunohistochemical diagnosis. While some studies evaluated changes in oxidative stress biomarkers in saliva, only a few studies have used animal models to additionally evaluate the histopathological changes after antioxidant therapy at the level of salivary glands [12,15,16].

Considering that it is difficult to obtain tissue for routine histopathological and biochemical analysis from human salivary glands, the effects of oxidative stress and antioxidant substances on humans are difficult to determine. However, strong evidence comes from experimental studies on laboratory animals, considering the interspecies high degree of similitude. The aim of this study is to find out the oxidative stress-induced changes in the salivary glands in experimental animal models after the administration of different substances and to evaluate their use as antioxidative treatment opportunities. The question is if the substances with antioxidant effects have the potential to reduce or reverse the effects of oxidative stress at the salivary gland level. In order to respond to this question, the main objective of the present review was to find histopathological data at the level of salivary glands supporting the hypothesis of the reversal of oxidative stress-induced changes after treatment using substances with an antioxidant effect.

## 2. Materials and Methods

A research question was on which of the substances with an antioxidant effect have the potential to reduce or reverse the effects of oxidative stress at the salivary gland level. The PICO format is as follows:

- P: oxidative stress;
- I: drug (chemotherapy, ethanol, heavy metals, fluoride, and other antioxidant substances);
- C: compared with control;
- O: animal outcome.

Data collection was conducted by searching the databases Pub Med, Science Direct, and Springer for articles published in the last 10 years, from 1 June 2013 up to the 1 June 2023, using a search strategy following the Preferred Reporting Items for Systematic Reviews and Meta-Analyses (PRISMA) [17]. There were 109 results on PubMed, 385 on Science Direct, and 1173 on SpringerLink.

### 2.1. Electronic Searches

The literature was searched independently by two groups of authors (L.B.U. and R.Ș.S. as well as C.M.G. and I.G.) using keywords and Boolean search terms defined in Table 1 to screen studies and to select full-text research articles. Moreover, hand-searching of relevant papers from all the references of included articles was achieved. Disagreements regarding the selected articles were resolved by discussion in the presence of a third group of authors (C.A., G.L.G., and C.G.P.) that acted as arbitrators when an agreement was not reached.

### 2.2. Study Selection

Research articles (full text) on oxidative stress-induced alteration in the salivary glands produced by drugs and other chemical substances, in experimental models, and reversal effects of antioxidants reflected in the histopathological features of salivary glands were included. The articles in English language were selected. The articles that were not available in full text and reviews were excluded from our search.

Outcomes were represented by the oxidative stress histological aspects induced by drugs or other chemicals and oxidative stress biomarkers, drug/chemical dose, route of administration, type of animal model, and administered antioxidants. We excluded studies that did not share the aforementioned characteristics through a full-text evaluation.

## 3. Results

### 3.1. Article Selection and Structure

**Figure 1:**
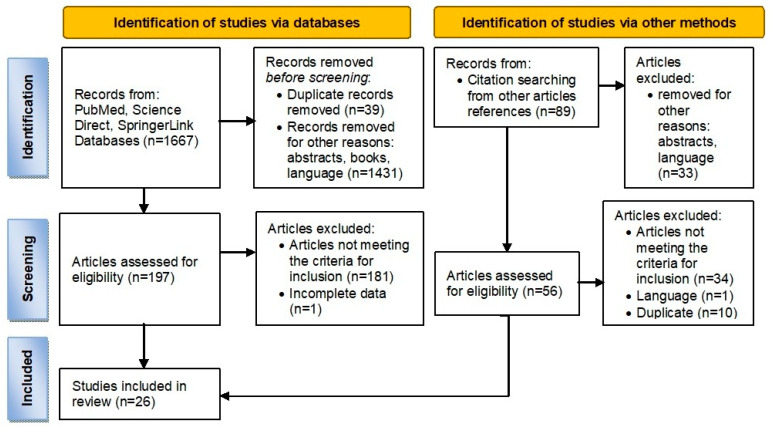
PRISMA flowchart.

Out of 1756 articles, 26 articles regarding the effect of different substances on salivary glands were included in the analysis (Figure 1). Their distribution was:

- 6 articles on the effect of chemotherapy drugs on salivary glands [5,12,15,16,18,19];
- 4 articles on the effect of ethanol on salivary glands [6,7,9,20];
- 9 articles on the effects of heavy metals on salivary glands [2,11,21,22,23,24,25,26,27];
- 3 articles on the effect of fluoride on salivary gland [4,10,28];
- 4 articles on the effect of other substances, such as atrazine [29], high protein diet [8], hyperoxia [30], and sodium nitrite [31];
- All included studies were case-control studies. The two types of laboratory animals used were rats and mice.

### 3.2. Drug-Induced Oxidative Stress on Salivary Glands in Animal Models

Only six studies have analyzed the effect of chemotherapy drugs on salivary glands, three of them using both histopathological and biochemical markers [15,16,18], two studies performed only histopathological analysis [12,19], and one conducted only biochemical evaluation [5].

According to these studies, drugs that produce oxidative stress-induced histological lesions at the level of the salivary gland and changes in oxidative stress biomarkers are antineoplastic drugs (cyclophosphamide and 5-fluorouracil), antibiotics (metronidazole), and analgesic drugs (tramadol).

The effects of these substances on the salivary gland were evaluated in experimental case-control studies on different strains of animals and are shown in Table 2. Supplemental File Table S2 presents other details regarding sample size and histopathology staining techniques used on the studies.

#### 3.2.1. Anticancer Drugs

Anticancer drugs induce cell death via oxidative stress, by the succession of the following processes: ROS induction–oxidative stress–apoptosis [12].

- Cyclophosphamide (CP), an alkylating agent used for the treatment of cancers and as an immunosuppressive drug for the treatment of autoimmune disease and in the case of transplants [12,15], affects a lot of organs, including the salivary glands, where it determines xerostomia due to decreased saliva production. The effect continues for a long time even after cessation of therapy. Two studies have analyzed the oxidative and morphological effects of CP on the salivary gland, one conducted on the PG and the other on the SMG. In the first study, the authors showed the oxidative impact on the PG by the increased level of oxidative markers and the decrease in antioxidants that were ameliorated by the administration of hesperidine [15]. The second study has not analyzed the biochemical markers [12]. Morphological analysis has shown vacuolar degeneration in both the PGs and SMGs [12,15], parenchymal atrophy, acinar apoptosis or necrosis, periductal edema, and fibrosis at the level of the SMG [12]. In the PG, there was acinar distortion, periductal inflammation, confirmed also by an increase in inflammatory markers, such as TNF-α and IL-1β, and a reduced Ki67 immunopositivity in acinar cells [15]. In the SMG gland, there was parenchymal atrophy, necrosis, and apoptosis, the latter being indicated by BCL-2 (B cell lymphoma 2) negativity, along with periductal edema and fibrosis [12]. Co-administration of hesperidine in the case of the PG gland [15] and selenium in the case of the SMG has diminished the alterations [12];
- 5-Fluorouracil (5-FU), a pyrimidine analog with cytostatic activity, used for the treatment of different solid tumors, especially from the genital tract, could induce salivary gland atrophy and xerostomia. Studies from the literature have shown that 5-FU can provoke oxidative stress by inhibiting the antioxidative system and by increasing the activity of the oxidative system. The lesions at the level of the PG could induce inflammation, apoptosis, and patchy necrosis of the epithelium and increase the number of myoepithelial cells. Morphological and biochemical changes were restored to normal after the administration of febuxostat [16].

#### 3.2.2. Metronidazole

Metronidazole, a chemotherapeutic drug also frequently used, considering its antimicrobial effect, for the treatment of oral cavity and dental pathologies, demonstrated that it could have a mutagenic effect by ROS inducement [5]. Only one study analyzed the oxidative stress effect on the salivary gland, showing an increase in the oxidative markers and a decrease in antioxidant defense in both the PG and SMG, with no significant difference between them. However, the morphological changes have not been investigated. There was a significant difference between the PG and SMG in GPx and CAT (*p* < 0.001) [5].

#### 3.2.3. Tramadol

Tramadol, a synthetic codeine analog [18] used as an analgesic drug [19], could also induce xerostomia due to a decrease in salivary secretion by 75% [18]. Two studies have shown the effect of tramadol on the PG [18] and on the SMG [19]. MDA increased in PG, beginning with 20 days from administration [18]. Morphological examinations have revealed almost the same changes (apoptosis, cytoplasmic vacuolization, and loss of basal position of nuclei) in both glands [18,19] with a few differences, e.g., a decrease in zymogen granules in parenchymal cell cytoplasms of the PG [18].

### 3.3. Chemical-Induced Salivary Gland Oxidative Stress in Animal Models

#### 3.3.1. Oxidative Stress Changes Induced by Ethanol Consumption in the Salivary Glands

Following our search, we found four articles on the effect of ethanol on salivary glands [6,7,9,20]: a study has analyzed the effect only from the histopathological point of view [24], four studies have evaluated both histopathological and biochemical parameters [11,21,22,23], two of which have been written by the same author group, being conducted in different experimental conditions [11,21]. The last four studies evaluated only the biochemical changes [2,25,26,27].

Effects of alcohol chronic consumption on salivary glands were evaluated on laboratory animals of different ages: adults [9], adolescents [6,7], and offspring laboratory animals [20]. The experiments were performed with different doses and periods of administration, followed by variable salivary gland lesions, depending on the age of the subjects [6,7,9,20].

The differences between different doses of alcohol at various ages, after acute or chronic alcohol consumption, are shown in Table 3. Histopathological and immunohistochemical analysis have shown only a few changes (Table 3), such as atrophy and fibrosis of the SMG gland in offspring. Ethanol administration in pregnant rats caused oxidative stress in the offspring’s PG, with increased MDA and NO levels [20]. Alcohol could induce oxidative stress in salivary glands of offspring and adolescent rats [7,20], by increasing MDA and NO up to two fold (Table 3). Mature rats presented only a low-level intensity of biochemical changes, indicating that the adults are more resistant to long-term alcohol consumption [9]. One-month ethanol administration by gavage determined an increase in alkaline phosphatase (ALP) in myoepithelial cells of salivary glands [9]; two months later, parenchymal atrophy with apoptosis and an increased proportion of the stromal area occurred [6]; four months later, inflammation and mast cell degranulation occurred; and six months later, increased acini NADPH was noticed [9]. Supplemental file Table S3 presents other details regarding sample size and histopathology staining techniques used in the studies.

Two studies on the PG of rats treated with ethanol during adolescence showed parenchymal atrophy, associated with a decreased immunopositivity of CK18 and CK19 in epithelial cells [6,7] and a decreased number of myoepithelial cells, while decreased immunopositivity for α-SMA was registered only in adolescent rats chronically treated with ethanol for 55 days [6]. In the SMG of adolescent rats, there was an increased stroma and acinar cell apoptosis after four weeks of ethanol administration [7] and ductal proliferation after almost two months of administration, evidenced by increased CK19 positivity in these cells [6].

In a study on male mature rats, ethanol administration had only a few effects after 30 days of administration, represented by an increased ALP (alkaline phosphatase) activity in acinar myoepithelial cells, in both types of glands highlighted by cytophotometry. After a prolonged administration of ethanol, for 120 days, tissular changes were noticed, such as lipid accumulation and parenchymal atrophy, with increased stroma proportion in both glands, along with PG inflammation and SMG mast cells degranulation [9].

The biochemical analysis was conducted only in the case of offspring and adolescent rats and revealed a significant increase in MDA and a significant decrease in ACAP in PG, along with a significant increase in MDA and NO in the SMG, in offspring rats [20], while MDA was both increased in the case of the PG and SMG in adolescent rats [7].

#### 3.3.2. Oxidative Stress Changes in the Salivary Glands Induced by Heavy Metals

Following our search, we found nine articles regarding the effects of heavy metals on salivary glands [2,11,21,22,23,24,25,26,27]. Heavy metals are toxic substances that can induce oxidative stress on the oral cavity and salivary glands. These metals are represented by aluminum (Al) [11,21], mercury (Hg) [22,24,25], lead (Pb) [23], and cadmium (Cd) [2,26,27].

The histological and biochemical effects of heavy metals on salivary glands are described in Table 4. Supplemental file Table S4 presents other details regarding sample size and histopathology staining techniques used in the studies.

Different concentrations of aluminum chloride (AlCl_3_) were administered for 60 days, inducing parenchymal atrophy and fibrosis in both the rat PG and SMG with a decrease in the SMG ductal area [21], while an increased stroma and atrophy of both acinar and ductal cells was registered in mice SMGs [11]. Biochemical changes were analyzed only in rats, the authors demonstrating the oxidative effect of AlCl_3_ [21]. None of these studies have evaluated the effects of antioxidant therapy [11,21].

Methylmercury (MeHg) administration in pregnant rats induced the following histological changes in the salivary glands of offspring rats: acinar atrophy and shrinkage of striated ducts are indicated by decreased CK19 and α-SMA immunopositivity, increased stroma in both the PG and SMG, and an increase in PG ductal area [24]. MeHg administration in male adult rats determined the increase in MDA in the PG and MDA and nitrite in the SMG [25].

Mercury chloride (HgCl_2_) administration in male adult rats induced histological changes in PG, with increased parenchyma and decreased stroma, decreased CK19 immunopositivity, and increased MT I/II positivity, while biochemical analysis has shown increased pro-oxidative markers (MDA and nitrite) in the SMG and decreased antioxidative ones (ACAP) in both the PG and SMG [22].

Pb administration, in doses of 50 mg/kg in male rats, confirmed the oxidative stress changes while the only morphological difference between glands was that of parenchymal atrophy in the SMG versus increased ductal areas in the PG [23].

Cadmium (Cd), found in cigarette smoke, could induce damage to different tissues, including salivary glands, by its oxidative properties [2,26,27]. There are no histopathological evaluations regarding cadmium’s effect on salivary gland tissues but biochemical evaluations have shown that Cd could induce oxidative stress with minor differences between studies, depending on the doses and periods of administration. A study was performed on the PG [27] and on another two studies on the sublingual gland (SLG) [2,26]. In two of them, the authors administered black chokeberry fruit as an antioxidant [2,27]; in the other one, zinc was administrated [26], restoring the oxidative status prior to injury in all cases. It was shown that when it is administrated to rats, in food and drinking water, both CAT and GPx decreased at the concentrations of 1 mg/kg for 3 months and 5 mg/kg for 10 months; in the SLG [2], SOD decreased in the PG after administration of a dose of 1 mg or 5 mg/kg for 10 months, while TOS, OSI, and PC increased after administration of a dose of 1 mg or 5 mg/kg for 10 months in the PG [27] and of a dose of 5 mg/kg for 10 months in the SLG [2].

#### 3.3.3. Oxidative Stress Changes in the Salivary Glands Induced by Fluoride Exposure

Following our research, we found three articles (Table 5) regarding the effects induced by fluoride on salivary glands [4,10,28]: one of them evaluated both histopathological and biochemical parameters [10] while two of them studied only the biochemical changes [4,28]. A study has analyzed the oxidative and antioxidative biochemical markers, both in plasma and tissue homogenate, after the administration of a high-protein diet. In the high-protein diet group, total antioxidant status (TAS) was 11 times higher in the PG than in plasma and 18 times higher in the SMG [8]. The total oxidant status (TOS) was lower with 99% in the PG and with 89% in the SMG in comparison with plasma. The oxidative status index (OSI) was 99.90% lower in the PG and 99.51% lower in SMG compared with plasma [8]. Supplemental file Table S5 presents other details regarding sample size and histopathology staining techniques used on the studies.

Fluoride, administered in pregnant Wistar rats in doses of 10 mg/L in drinking water, decreased TEAC both in the PG and SMG of offspring while a dose of 50 mg/L in drinking water increased TEAC in the PG of offspring and decreased TEAC in the SMG of offspring. The morphological examination and CK18 immunostaining showed no differences between offspring groups regarding the parenchymal and stromal areas but the exposure to higher fluoride concentration increased the ductal area in both glands [10]. Thus, higher doses of fluoride in pregnant Wistar rats induced higher total antioxidant capacity in the PG of offspring and lower antioxidant capacity in the SMG of offspring [10]. On the other hand, fluoride administered in male Swiss Albino rats in doses of 10 mg or 50 mg F/L in drinking water determined the GSH increase in the SMG [28]. When 15 mg F/kg was intraperitoneally administrated in male Wistar rats, there was an increase in MDA and a decrease in CAT in the PG and an increase in MDA and CAT in the SMG, while SOD decreased after 1 h, increased after 3 h, and it then decreased again after 24 h in the SMG. Higher fluoride doses induced more pronounced oxidative stress in the SMG than in the PG [4].

#### 3.3.4. Oxidative Stress Changes in the Salivary Gland Induced by Other Substances

- Effects of atrazine on salivary glands. Atrazine is a herbicide that is toxic for many tissues and induces oxidative stress in fish and laboratory animals. A study performed by Ahmed et al. in 2022 on adult male albino rats that were exposed to atrazine showed, by biochemical and molecular analysis, a decreased level of GSH and an increased level of MDA [29]. The real-time polymerase chain reaction analysis has shown an increased expression of the following genes: TNF-α (tumor necrosis factor-alpha), BCL-2 (B cell lymphoma 2), Creb1 (cAMP-responsive element binding protein 1), and Drd1 (dopamine receptor D1) in the salivary glands of rats exposed to atrazine. Histopathological examination showed vacuolar degeneration of acinar cells, intraductal epithelial cells shedding as a sign of toxicity, and vascular congestion. The immunohistochemistry has shown caspase-3 strong immunopositivity in the SMG of the exposed rats [29];
- High-protein diet-induced oxidative stress. In a study performed by Kolodziej et al. in 2017 on male Wistar rats that received a high-protein diet, the authors showed that SOD decreased only in the SMG and TAS increased only in PG, while TOS and OSI increased in both glands [8];
- Hyperoxia-induced oxidative stress. Tajiri et al. in 2019 conducted a study on male mice exposed to hyperoxia and showed that hyperoxia induces oxidative stress but not inflammation, with the SMG being more sensitive to this type of stress [30];
- Effects of sodium nitrite on salivary glands. In a study conducted by Elsherbini et al. in 2020 on rats, it was shown that sodium nitrite could increase the MDA level and also decrease GSH and TAC. The main morphological changes are represented by inflammation, fibrosis, and discontinuity of the myoepithelial layer that surrounds the acini epithelium in the SMG [31].

### 3.4. Antioxidants with Restorative Effects on Salivary Glands Changes Induced by Oxidative Stress

There are many antioxidants (vitamins, polyphenols, hesperidin, and oligoelements, such as selenium, zinc, etc.) but only some of them were evaluated for their effects on oxidative stress changes in salivary glands. The antioxidant substance has been administered before the toxic substance in four studies [12,15,16,31] and co-administration in four studies [2,19,26,27]. A summary of their effects on histological or oxidative biomarkers on salivary glands is shown in Table 6. 

Hesperidin (HSP), a natural product with an antioxidative role successfully used as a treatment against chemotherapy toxicity, restored the biochemical markers modified by cyclophosphamide (CP) administration on male albino rats’ salivary glands [15], while selenium (Se) reduced the damage induced by chemotherapy, as was shown by histology exam results, showing just minimal morphological alterations represented by mild degeneration of epithelial cells and vascular congestion, in male adult albino rats [12]. Hesperidin also has an anti-inflammatory effect, by reducing TNF-α and IL-1β [15]. Ki67 and α-SMA immunohistochemistry have shown a moderate increased expression in the coadministration of CP and hesperidin, compared with the CP group, indicating the protective effect of hesperidin against CP action [15]. Thus, coadministration of CP and hesperidin or Se may reduce the damage induced by chemotherapy [12,15].

Selenium is a widely distributed element in food of vegetables (fruits, cereals, and vegetables), in food of animal origin (meat and milk), and also in multivitamin supplements that have antioxidative properties, by reducing free radicals. Its coadministration with chemotherapy could reduce the tissues’ oxidative damage [12].

Febuxostat is a drug used for the treatment of gout that has anti-inflammatory properties, by inhibiting TNF-α and IL6, along with antioxidant and antifibrotic roles. It was shown that the coadministration with 5-Fluorouracil led to a decrease in the level of MDA and NO together with an increase in SOD and GSH [16]. Histopathological findings and immunohistochemical reactions have shown only slight changes, such as mild acinar vacuolation and a moderate α-SMA immunostaining [16].

Ginger (*Zingiber officinale*) has anti-inflammatory and anti-oxidant effects, being also involved in wounds healing. 10-Dehydrogingerdione (10-DHGD), a compound extracted from *Zingiber officinale*, has anti-inflammatory and antioxidant effects and seems to be involved in tissue regeneration. Analysis of the SMG after coadministration of 10-DHGD with tramadol in male albino rats (Wistar) has shown that caspase-3 positivity became lower compared with tramadol group and the histopathological aspect was almost normal, with PAS (periodic acid Schiff) positivity of acinar and ductal cells, showing that cells contain normal zymogen granules. Thus, it is demonstrated that coadministration of 10-DHGD has a protective effect against the oxidative stress and apoptosis induced in the SMG by tramadol [19].

Black chokeberry fruits are rich in antioxidants, such as proanthocyanidins and anthocyanins, and can prevent oxidative damage at the level of the parotid gland, the effect being correspondingly greater in case of a longer duration of administration [2,27].

Zinc is thought to reduce the digestive level of cadmium and also have antioxidative properties. Studies from the literature have shown that zinc could protect the salivary gland only at higher concentrations and after a long period of administration [26].

Glycyrrhizic acid (GA) is extracted from *Glycirrhiza glabra*, a type of licorice root. The biochemical analysis of the rat salivary gland tissue to evaluate the GA effect on injuries induced by sodium nitrite (SN) has shown that the levels of MDA, GSH, TAC, TNF-α, and IL-1β were restored after the administration of GA. The histological examination has shown that in the SN group, the area around the salivary gland acini reacts positively with CD68, a marker for macrophages, and with α-SMA. PAS reaction was used to check the continuity of basement membranes of salivary gland acini, showing its discontinuity in the SN group. The administration of GA restored the normal architecture of salivary glands [31].

## 4. Discussion

Following our search, we found 26 articles with data on tissue homogenate used for biochemical analysis of oxidative and antioxidative markers, hematoxylin eosin (HE), and immunohistochemical analysis used for histopathological diagnosis in salivary glands. The most interesting observation of our study was that some substances, such as the pre-administration of glycyrrhizic acid prior to the administration of sodium nitrite, had the effect of restoring normal tissue architecture, with restoration of the basement membrane continuity of the acinar epithelium [30]. We observed from our review of these articles that the prior administration of hisperidine in the case of cyclophosphamide [15] and glycyrrhizic acid in the case of sodium nitrite [31] had an anti-inflammatory effect [15,31], while coadministration of 10-DHGD with tramadol had an antiapoptotic effect [19].

Oxidative stress-induced changes in other tissues are well described, considering that the loss of balance between the generation of ROS and antioxidant capacity is a characteristic of many pathological processes [32]. In recent years, studies have proved the involvement of oxidative stress in different pathologies, like cardiovascular, kidney, neurodegenerative, pulmonary, and malignant diseases, as well as in the process of aging [33].

To our knowledge, there are very few studies that address the effects of oxidative stress on salivary glands in humans and their connection to pathology, as follows: two studies on oxidative stress markers in patients with PG tumors [34,35], a review that deals with the diseases that impair the salivary function and their treatment [36], an article regarding the oxidative markers in HIV patients [37], seven studies on cell cultures from human salivary glands [38,39,40,41,42,43,44], and some studies about salivary gland damage by oxidative stress in systemic diseases, such as arthritis [45,46], Alzheimer’s disease [47], stroke [48], Sjogren’s syndrome [49,50], chronic heart failure [51], psoriasis [52], and chronic kidney disease [53].

Although different in size and slightly different in location, rodents’ major salivary glands are similar to human counterparts, in morphological and functional terms. Thus, rodents are widely used in research, considering the facility in their acquisition and well-known ethical protocols. Three pairs of main salivary glands are present in both species but caution should be paid in dissection as the SMG and SLG are fused in mice. Parasympathetic and sympathetic innervation of the autonomic nervous system is responsible for saliva secretion regulation in both species, with thicker myelin sheaths and connective tissue nerve sheaths in humans. Additionally, blood irrigation has different routes for the two species [54].

Regarding their histology, the parenchyma and stroma are similar, lobular parenchyma being composed of acinar serous and mucous cells or seromucous and myoepithelial cells [55]. Moreover, intercalated ducts (cuboidal epithelium with stem cells), striated ducts (columnar epithelium with basal infoldings containing mitochondria), excretory ducts (variable types of columnar cells), and main excretory ducts (named in humans as Stenon’s for the PG, Wharton’s for the SMG, and Bartholin’s or ducts of Ravinus for the SLG) are identified in both species, while capsule and septae delimitate the lobules, with intralobular adipose tissue in human PGs [54,55].

In both species, the PG is only composed of serous acini, with secretory cells; the SMG is a mixed gland in humans and pure serous in rodents, with no demilunes; and the SLG is a mixed gland in both species [55]. The ends of mucous acini are covered by serous demilunes due to chemical fixation in the SLG of both rodents and humans and in the SMG of humans [55].

However, due to rodents’ SMG sexual dimorphism (more prominent in rat SMGs compared to mouse SMGs), granular convoluted duct (GCT) cells are serous-like large exocrine cells situated between the intercalated and striated ducts of SMGs of male mice; secretory columnar epithelium, with hormones and cell growth factors, such as brain-derived neurotrophic factor (BDNF), epidermal growth factor (EGF), hepatocyte growth factor (HGF), insulin-like growth factor type 1 (IGF-1), nerve growth factor (NGF), transforming growth factor-α (TGF-α), and transforming growth factor-β (TGF-β); and pillar cells with paracrine function, containing fibroblast growth factor 2 (FGF2) or basic fibroblast growth factor (FGF). Whereas, granular intercalated duct cells are located in the intercalated ducts of female mice (representing remnants of the perinatal secretory cells phenotype of the terminal tubules of immature SMGs, with proliferative and differentiation capacities) [54,55]. Mainly due to the content of GCT cells, differences in histological, histopathological, and immunohistochemical features should be expected between rodents and human salivary glands. Moreover, non-specific immunohistochemical reactions of rodents’ salivary glands to secondary antibodies are remarkable in ductal epithelium, possibly due to the secretory immunoglobulin transcytosis process, which could lead to misjudgments in the interpretation of weak immunohistochemical staining, a negative control being recommended in this situation [55].

Functionally, saliva has a comparable composition in both species, facilitating the interpretation of results obtained in experimental models, represented by a solution containing proline-rich proteins, secretory immunoglobulin A (sIgA), immunoglobulins M and G (IgM and IgG), and amylase, produced by serous acini of PG; a viscous solution with mucins, produced by mucous acini of the SLG; and a mixed solution of sIgA, IgM, and IgG produced by mucous and mixed acini [55]. However, caution should be taken in translational salivary gland research, considering the interspecies histological, histochemical, and immunohistochemical differences.

**Figure 2:**
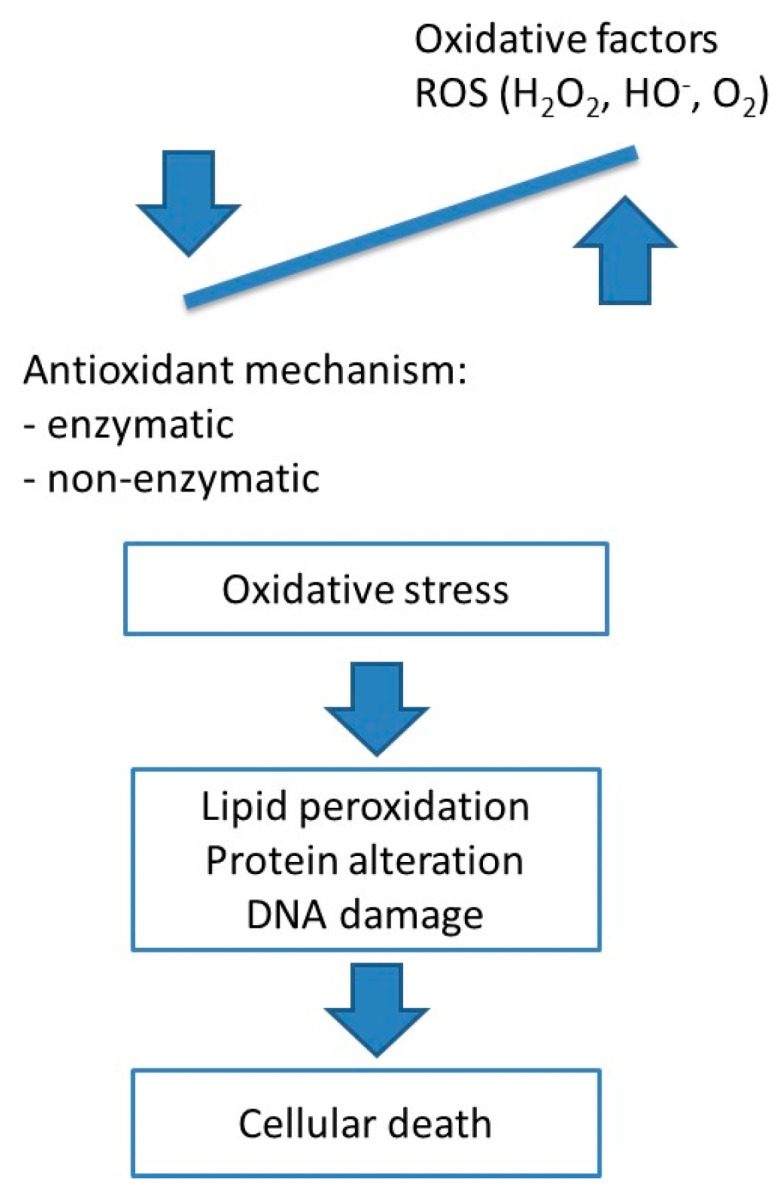
The imbalance between oxidant and antioxidant factors leads to oxidative stress. ROS: reactive oxygen species.

Due to increased ROS production at the level of salivary glands, oxidative stress is characterized by an imbalance between oxidants and antioxidants in favor of the former, resulting in the oxidation of lipids, DNA damage, and protein alteration that might lead to cellular death (Figure 2). The mechanisms involved in this process are not yet fully understood.

Damage to salivary glands can cause dysfunction leading to alterations in salivary flow and quality [55]. Decreased or absent salivation leads to periodontitis [20], while reduction in salivary secretion is characteristic of xerostomia [12]. Xerostomia is characterized by a dry mouth that affects chewing, swallowing, tasting, and speech, it induces pain, periodontitis, and bad breath leading to malnutrition, impaired social activities, and oral candidiasis [10,15,24,56]. Reduced CK18, CK19, and α-SMA expressions are associated with decreased saliva production and xerostomia [15,24,55].

The biochemical, regulatory, and secretory mechanisms used by the PG and SMG to react to harmful exposures are variable [23]. The decreased acinar area in both glands indicates the decline in saliva production. The ductal system’s functions include removing saliva from the oral cavity and replacing acinar cells that have been damaged [10]. The rate between parenchyma and stroma favors the stroma indicating a tendency to connective type repair and fibrosis in salivary glands [21]. Nevertheless, parenchymal atrophy of salivary glands associated with fibrosis and inflammation are signs of sialadenitis. Moreover, salivary glands’ cellular population can change their phenotypes towards acinar, ductal, and myoepithelial cells [56].

The treatment with antioxidants could be a future opportunity either individually or as an adjuvant therapy with the aim of preventing the complications of oxidative stress. The studies that use antioxidants are even fewer than those of oxidative damage on salivary glands. Additionally, a cut-off value for oxidative stress and antioxidant markers for the indication of antioxidant therapy is not yet established.

Drugs used in cancer therapy, such as 5-FU and CP, can cause oxidative stress leading to xerostomia and inflammation (mucositis) due to increased levels of proinflammatory cytokines associated with periductal edema and apoptosis, clinically manifested by swelling and pain [12,57]. Anticancer drugs produce acrolein, a metabolite that causes oxidative stress by producing ROS and NO, which damage to intracellular lipids, proteins, and DNA, thereby preventing cell division and stimulating apoptosis [12,15].

Our search identified two studies with hesperidin and selenium that can reverse the damage induced by CP. Ki67 immunostaining showed a strong positivity after hesperidin administration concomitant with CP that indicates the epithelial cells’ capacity to regenerate following CP-induced salivary glands injury by inflammation and oxidative stress [15]. Much more, BCL-2 was used to show that selenium prevents cell apoptosis when administered concomitant with CP. Selenium, included in multivitamins products, has been demonstrated to have a protective effect against oxidative damage of salivary glands induced by radiotherapy [12]. CP could induce changes in both the acinar and ductal epithelium, with apoptosis or necrosis and vacuolar degeneration [12,15] occurring by a direct cytotoxic effect which, in combination with stromal fibrosis, results in an increased stromal area [12]. Continued CP administration induces the formation of ROS and the release of pro-inflammatory cytokines, including IL-1β, TNF-α [15], etc. CP inhibits DNA replication and induces apoptosis [15,58] via the caspase pathway [58]. Moreover, decreased α-SMA expression indicates the reduction in myoepithelial cell function in CP administration. Myoepithelial cell function is achieved by their intermediate filaments that contract in order to release saliva from acini into salivary ducts [10,21,54]. They are also important for extracellular matrix synthesis [54]. Considering these accumulated data, myoepithelial cell decreases could explain the reduced saliva production in cancer therapy [10].

Our search found an experimental study, with 10-dehydrogingerdione (10-DHGD), a compound extracted from ginger, that has a protective effect against the oxidative stress and apoptosis induced by tramadol. Tramadol can cause xerostomia [19]. Histological evaluation after tramadol in high doses showed vacuolar degeneration and apoptosis in the PG due to mitochondrial damage [18]; alternatively, vacuolar changes can also result from severe damage to the endoplasmic reticulum [59].

There are no experimental studies associated with the histopathological evaluation of the antioxidant effect on salivary glands in the case of other types of drug administration. 5-FU could also induce cell necrosis by a direct cytotoxic effect, inflammation evidenced by the increased levels of TNF-α and IL-1β that have been attributed to oxidative reactions and to an attempt at myoepithelial cells’ regeneration [16]. Metronidazole metabolites have the potential to cause DNA damage or ROS generation, making them potentially genotoxic. It has been already demonstrated that ROS generated during antibiotic therapy play a significant role in bactericidal activity but they are also mutagenic [5].

Ethanol, tobacco, heavy metals, fluoride, and other chemicals cause oxidative stress, which determines the release of proinflammatory cytokines and changes in salivary flow and composition, leading to xerostomia [2].

There are no experimental studies associated with histopathological evaluation of the antioxidant effect on salivary glands after acute or chronic ethanol consumption, with heavy alcohol drinking being a worldwide health issues. It is well known that chronic alcohol consumption leads to salivary gland atrophy [6,9] with consequent salivary production reduction [6,20] and salivary gland damage [6,9,20]. Immediately after intake, alcohol can be found in saliva [6]. Chronic ethanol administration affects PGs more severely and for a longer period of time, by decreasing the number of myoepithelial cells and by lipid peroxidation induction [7]. Morphological changes due to the atrophy of PG parenchyma may occur following both acute or chronic drinking, while the increased fibrous stroma and apoptosis appear only after a chronic consumption of at least four weeks, in the SMG [6,7]. It is possible that the PG is able to gradually adapt to chronic ethanol intoxication, whereas the SMG is more resistant to short-term exposure. However, in the case of administration of ethanol for a long period of time (more than 120 days), lipid metabolism disorders may occur, morphologically expressed by intracellular and stromal lipid accumulation, in both the PG and SMG, inflammation and vascular congestion in PG, and mast cells degranulation in the SMG [9]. Chronic ethanol ingestion, depending on the dose and duration, causes parenchymal loss and stromal edema and fibrosis, leads to sialadenitis, while fatty infiltration is due to improper lipid metabolism [9]. Mast cell degranulation may occur as a result of a hypersensitivity reaction induced by oxidative stress but more studies are needed to demonstrate this hypothesis [9]. Cellular vacuolation may be also the consequence of organelles damage [12]. Moreover, changes in the microvasculature of ductal cells can also occur. Heavy ethanol drinking induces oxidative stress in both the PG and SMG [20]. Chronic alcoholism increases the risk of dental diseases as well as periodontitis due to decreased salivary flow and altered protein and electrolyte levels [6,9,20]. Another current issue is that alcohol consumption has increased among teenagers [7]. Ethanol exposure during adolescent ages causes parenchymal atrophy in PGs and apoptosis in SMGs [6], leading to earlier and more extensive salivary glands lesions and their spectrum of complications.

Low heavy metal exposure is difficult to be diagnosed, compared to intoxications. However, there was no study associated with the histopathological evaluation of antioxidants’ effect on salivary glands after exposure to heavy metals revealed by our search. The SMG and PG react differently to metal exposure [23], considering that the PG becomes more susceptible to toxic substances due to increased protein synthesis [20].

Mercury exposure may cause stomatitis [22]. The cytoskeletal integrity of acinar and myoepithelial cells can be harmed by exposure to metals like aluminum and mercury, which can also cause volumetric changes and cell death [22,23,25,56]. Hg, even in low doses, can lead to salivary lesions. Only SMG changes have been reported in Hg exposure [22]. MeHg can cause xerostomia in populations at high risk of environmental exposure [56]. MeHg crosses the placenta and affects fetal development [24]. The difference between glandular changes after administration of MeHg was attributed to the increased affinity of mercury compounds to the sulfhydryl proteins from the composition of saliva produced by the PG [25]. The authors of the study concluded that the parenchymal atrophy could have induced a decrease in saliva production and xerostomia in the case of pre and postnatal MeHg exposure [25]. The same authors have shown nitrite increases in the SMG. This was attributed to the increased production of ROS in the SMG [24,25]. In mercury poisoning, the oxidative damage is so severe that tissues lose their ability to repair and even undergo apoptosis and cell death [25].

AlCl_3_ exposure contributes to inflammation and oxidative stress [11] due to its pro-oxidant properties [11,21]. The SMG is susceptible to oxidative stress caused by low-dose and long-term systemic AlCl_3_ exposure. Experimental studies conducted on animals that received AlCl_3_ showed an increased SMG ductal area in rats, while atrophy of ductal areas and increased stroma were registered in mice SMGs, possibly due to different doses administered or due to them being different animal species [11].

Lead can cause cellular, morphological, and biochemical damage to the PG and SMG as well as promotion of glandular oxidative stress. The presence of intracellular Pb affects the microfilaments of the myoepithelial cell’s skeleton, which are crucial for PG contraction and saliva secretion, as evidenced by the decreased CK-19 immunopositivity in both the PG and SMG, explaining the decrease in saliva flow. Additionally, ductal atrophy may also affect saliva production [23].

Even low levels of chronic exposure to cadmium (Cd) harm the oral cavity and can cause neoplastic and non-neoplastic diseases, like oral pigmentation, dysphagia, and a diminished capacity for soft tissue regeneration [27]. Passive exposure to cadmium from smoke can contribute to the development of dental caries and periodontitis due to decreased enamel mineralization [26]. Decreased or absent salivation leads to periodontitis [20]. Long-term exposure to Cd from smoking can damage to the SLG [26]. Compared to the other two major salivary glands, the SLG is less vulnerable to oxidative stress induced by Cd. Cd-induced oxidative stress causes oxidative damage to lipids, proteins, and DNA. It was demonstrated that Cd alters heme degradation, cell proliferation, and differentiation, possibly causing apoptosis or necrosis, added to the alteration of cell membrane permeability, by phospholipid damage [2].

Fluoride is widely used but there are no experimental studies associated with the histopathological evaluation of the antioxidants effect on salivary glands after fluoride excessive exposure. The use of fluoride in toothpaste, food, and water could increase the quantity of fluoride exposure, having, as a consequence, the oxidative stress and morphological changes at the level of teeth and salivary glands [4,10]. Even if it is used to treat the teeth decay, fluoride can pass through the cell membrane and affect different tissues, such as the salivary glands, where it can impair the action of antioxidant systems [4]. Fluoride exposure may cause mitochondrial damage, which would then lead to apoptosis [10]. The SMG is more susceptible to oxidative stress and inflammation in case of fluoride chronic administration or perinatal exposure [10,28]. Offspring are four times less affected than adults in terms of the severity of lesions [10]. SOD variation in the SMG after the administration of fluoride to male rats was attributed by the authors to a transient adaptive response [4]. All these changes have confirmed the presence of oxidative stress induced by fluoride administration. Since the PG and SMG have different metabolisms, the authors have shown that fluoride intoxication induced more pronounced oxidative stress in the SMG than in the PG [4].

Our search found an experimental study with glycyrrhizic acid that restored the injuries after the administration of SN. Chronic exposure to SN induces inflammation and oxidative stress by free radical production, lipid peroxidation, and a reduction in endogenous antioxidant defenses [31]. Inflammatory tissue lesions induced by SNs are due to macrophage recruitment and cytokine release [31]. Chronic exposure to SN is also mutagenic and carcinogenic [31].

High protein intake has a role in oxidative stress induction [8]. Moreover, atrazine affects DNA and can be involved in carcinogenesis [29].

**Figure 3:**
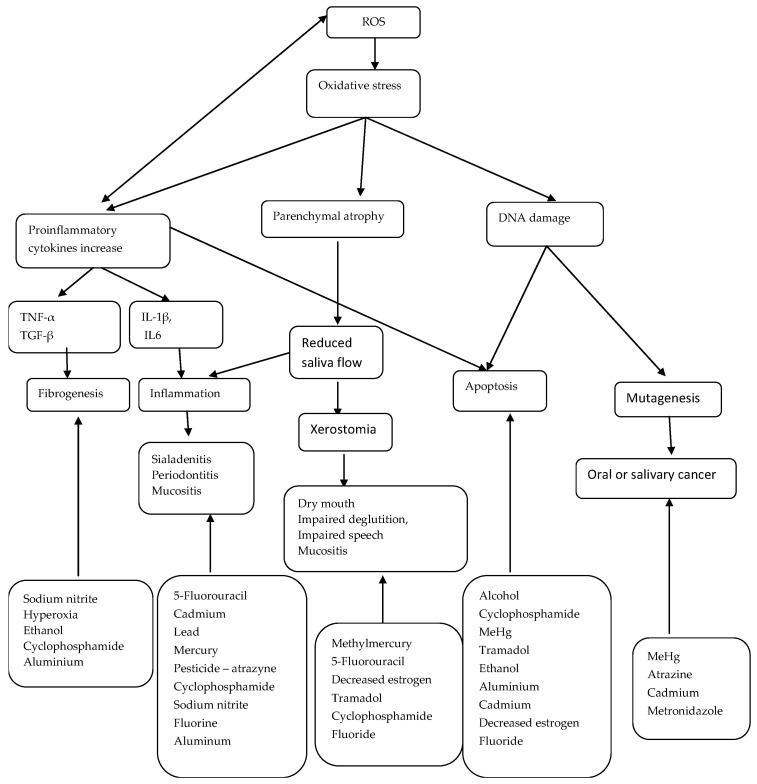
The effects of oxidative stress on salivary glands and inducing substances. TNF-α: tumor necrosis factor-alpha, TGF-β: Transforming growth factor beta, IL-1β: interleukin-1β, IL6: interleukin 6, DNA: Deoxyribonucleic acid

A schematic overview of the effects of drugs and other chemicals on the salivary gland is shown in Figure 3.

All the antioxidative treatment used so far has shown the recovery of normal previous architecture and functions, associated with a decrease in prooxidative markers. Recently, it was demonstrated that the salivary glands have the ability to change their cellular phenotypes as a response to metabolic disorders [56]. In light of the new discovery regarding the salivary gland stem cells’ property to differentiate toward different cell types (acinar, ductal, and myoepithelial) and their possibility to change their phenotype as a reaction to the environment [60], it can be concluded that stem cells may have the ability to restore the damaged salivary gland [61] but more studies are needed to evaluate the function of salivary stem cells in case of metabolic or oxidative stress disorders. All the presented data demonstrate that toxic substances induce complex salivary gland lesions, characterized by epithelial atrophy, stromal fibrosis, apoptosis, inflammation, and changes in lipid metabolism.

The knowledge of the anti-oxidant effects of different substances at the level of the salivary glands might be useful to reduce the toxic effects of chemotherapy or radiotherapy at this level. Moreover, the ability of some substances to restore the damaged salivary gland at the histological level might increase their advantage.

In humans, it is difficult to perform a biopsy after each administration of an antioxidant substance to see the effect. The limitation of animals is that the observation period is short.

## 5. Conclusions

Many substances, such as drugs (antineoplastic drugs, antibiotics, and analgesics), alcohol, heavy metals, and fluoride may increase oxidative stress, including in salivary gland tissues, resulting in morphological and functional changes, as follows: epithelial atrophy, stromal fibrosis, apoptosis, inflammation, and changes in lipid metabolism. Substances such as hesperidin, selenium, 10-dehydrogingerdione, and glycyrrhizic acid are anti-oxidants with proven restorative effects on salivary glands in damages induced by oxidative stress, after exposure to drugs and other chemical substances in rodents; but demonstrating their similar effects in human salivary glands is challenging. Since there are only very few studies in humans, the results obtained in experimental studies need to be validated in clinical studies. Further studies are needed to evaluate the antioxidant effects of new drugs on salivary gland levels. One direction might be to evaluate anti-oxidants useful to reduce chemotherapy toxicity at the level of salivary glands.

## Figures and Tables

**Table 1:** Search strategy.

Theme	Keywords and Boolean Descriptors
oxidative stress	(“Oxidative Stress”) OR (“Antioxidants”)
salivary glands and saliva	(“Salivary Glands” OR “Submandibular Glands” OR “Parotid Gland” OR “Sublingual Gland”)
histology	(Histology OR Ultrastructural OR Histopathology)

**Table 2:** Oxidative stress histological and biomarkers changes induced by drugs on salivary glands in experimental case-control studies.

Drug	Animals	Dose and Route of Administration Duration	Salivary Gland	Histopathological Staining Technique	Histological Aspects	Oxidative Stress Markers ^#^	Antioxidant Administered	References
Cyclophosphamide	Adult male albino rats	200 mg/kg Intraperitoneal Single injection in the 7th day of the experiment	PG	HE Mallory α-SMA ki-67	Inflammation: ↑ TNF-α, ↑ IL-1β Vacuolar degeneration Distortion of acini with nuclear pyknosis Ducts dilation Increased stroma ↓ α-SMA ↓ myoepithelial cells ↓ ki67	↓ CAT, ↓ GPx, ↑ MDA	Hesperidine	Mostafa et al., 2003 Egypt [15]
	Adult male albino rats	150 mg/kg Intraperitoneal Single injection in the 8th day of the experiment	SG	HE Bcl-2	Acinar atrophy or apoptosis Necrosis of acini, striated ducts, and granular convoluted tubules Vacuolar degeneration Peri-ductal edema Peri-interlobular ducts fibrosis		Selenium	Alnuaimi et al., 2002 Iraq [12]
5-Florouracil	Adult male albino rats	35 mg/kg Intraperitoneal From day 10 to 14	PG	HE Toluidine-Blue α-SMA	Inflammation: ↑TNF-α, ↑IL-1β Patchy necrosis of epithelium ↑ α-SMA ↑ acinar myoepithelial cells	↓ GSH, ↓ SOD, ↑ MDA, ↑ NO	Febuxostat	Abdelzaher et al., 2002 Egypt [16]
Metronidazole	Male Wistar rats	100 mg/kg in 0.5 mL drinking water 7 days	PG, SMG			↓ CAT, ↓ SOD, ↓ GPx, ↓ TAS, ↑ TOS, ↑ TOS/TAS ↑ LPO		Onopiuk et al., 2018 Poland [5]
Tramadol	Adult Albino rats	20 mg/kg Gastric tube 20 days	PG	HE Toluidine blue capase-3	Apoptosis Vacuolar degeneration Loss of nuclear polarity			Elhindawy et al., 2019 Egypt [18]
		20 mg/kg Gastric tube 30 days	PG		Apoptosis Vacuolar degeneration Zymogen granules in cytoplasm			
	Male Wistar rats	20 mg/kg Intraperitoneal 45 days	SMG	HE PAS caspase-3	Acinar and ductal lipid and vacuolar degeneration and apoptosis Loss of nuclear polarity		10-dehydroginger-dione from ginger extract	Hassabou et al., 2021 Egypt [19]

Bcl-2 (B cell lymphoma 2); CAT: catalase; Gpx: glutathione peroxidase; GSH: reduced glutathione; HE: hematoxylin eosin stain; IL-1β: interleukin-1β; ki67: a proliferation marker, LPO: lipid peroxidation; MDA: malondialdehyde; NO: nitric oxide; PAS: periodic acid Schiff; PG: parotid gland; α-SMA: smooth muscle actin; SMG: submandibular gland; SOD: superoxide dismutase; TAS: total antioxidative status; TNF-α: tumor necrosis factor-alpha; TOS: total oxidative state; ↓: decreased; ↑: increased. ^#^ only significant change vs. control: *p* < 0.05.

**Table 3:** Oxidative stress histological and biomarkers changes induced by ethanol exposure on salivary gland experimental case-control studies.

Animals	Dose Route of Administration Duration	Salivary Gland	Histopathological Staining Technique	Histological Aspects	Oxidative Stress Markers ^#^	References
Offspring rats	3.0 g/kg Gavage 3 days alcohol in pregnant rats and a period of 4 days of abstinence	SMG	HE CK-19 Vimentin	Parenchymal atrophy Stromal fibrosis	↑ MDA, ↓ ACAP	Ferreira et al., 2021 Brazil [20]
		PG		↓ parenchymal area, ↑ stromal area, ↓ acinar area, ↓ ducts	↑ MDA, ↑ NO	
adolescent female Wistar rats	3 g/kg/day Gavage 1 week	- PG	α-SMA, CK-18 Vimentin	- Parenchymal atrophy - ↓ CK18	↑ MDA	Fagundes et al., 2016 Brazil [7]
	3 g/kg/day Gavage 4 weeks	- PG		- Parenchymal atrophy - ↓ CK18	↑ MDA	
		- SMG		- Increased stroma - Apoptosis	↑ MDA	
	6.5 g/kg/day Gavage 55 days	PG	α-SMA, CK19 caspase-3	Parenchymal atrophy ↓ α-SMA ↓ CK19		Fernandes et al., 2015 Brazil [6]
		SMG		Increased stromal area ↑ CK19 in ductal cells Apoptosis in acinar and ductal area indicated by caspase 3 positive cells		
mature male Wistar rats	6.9 g/kg/day of 20% ethyl alcohol solution Gavage 30 days	PG, SMG	HE toluidine blue	↑ ALP in myoepithelial cells of terminal acini		Sorkina et al., 2022 Russia [9]
	6.9 g/kg/day of 20% ethyl alcohol solution Gavage 120 days	- PG		- Inflammation		
		- PG, SMG		- Parenchymal atrophy - Lipid accumulation		
		- SMG		- Mast cell degranulation - Edema		
	6.9 g/kg/day of 20% ethyl alcohol solution Gavage 180 days	SMG		↑ NADPH in acini		

ACAP: Antioxidant capacity against peroxyl radicals； ALP: alkaline phosphatase; CK18: cytokeratin 18; CK19: cytokeratin 19; HE: hematoxylin eosin stain; MDA: malondialdehyde; NADPH: nicotinamide adenine dinucleotide phosphate; NO: nitric oxide; α-SMA: smooth muscle actin; ↓: decreased; ↑: increased. ^#^ only significant change vs. control: *p* < 0.05.

**Table 4:** Heavy metals’ oxidative stress histological and biomarkers changes on salivary glands experimental case-control studies.

Metal/Metal Salt	Animal	Dose Route of Administration Duration	Salivary Gland	Histopathological Staining Technique	Histological Aspects	Oxidative Stress Markers ^#^	Antioxidant	References
AlCl_3_	male Wistar rats	8.3 mg/kg in DW Intragastric gavage 60 days	PG, SMG	HE	Fibrosis Parenchymal atrophy	↓ ACAP, ↑ LPO		Souza-Monteiro et al., 2022 Brazil [21]
			SMG		Increased ductal area			
	male Swiss albino mice	18.5 mg/kg in DW Intragastric gavage 60 days	SMG	HE	Atrophy of acinar and ductal area Increased stroma			Souza-Monteiro et al., 2020 Brazil [11]
			PG, SMG			↑ MDA, ↓ GSH		
MeHg	pregnant Wistar rats and their offspring	40 μg/kg Dissolved in ethanol and included in cookies Both gestational and lactation periods	PG, SMG	HE	Acinar atrophy Smaller striated ducts Increased stroma ↓ CK19 ↓ α-SMA			Souza-Monteiro et al., 2022 Brazil [21]
			PG		Increased ductal area			
	male Wistar rats	0.04 mg/kg, diluted in corn oil 35 days	PG, SMG			↑MDA		Farias- Junior et al., 2017 Brazil [25]
			SMG			↑Nitrite		
HgCl_2_	male albino rats	0.375 mg/kg, Intragastric gavage 45 days	SMG	MT–I/II CK19	Increased parenchyma Reduced stroma	↑ MDA ↑ Nitrite		Aragão et al., 2017 Brazil [22]
			PG, SMG		↓ CK19, ↑ MT I/II	↑ ACAP		
Pb	male Wistar rats	50 mg Pb/kg/day Intragastric gavage 55 days	PG, SMG	HE MT I/II α-SMA	↑ MT I/II, ↓ α-SMA	↑ MDA		Lopes et al., 2020 Brazil [23]
			PG		Increased duct area	↑ Nitrite		
			SMG		Parenchymal atrophy			
Cd	Female Wistar rats	5 mg/kg Food 3 months	PG			↑ LPO	Black chokeberry fruit	Dąbrowska et al., 2019 Poland [27]
		1 mg or 5 mg/kg Food 10 months				↓ SOD, ↑ TOS, ↑ OSI, ↑ PC		
	Female Wistar rats	1 mg/kg Food 3 months	SLG			↓ CAT, ↓ GPx	Black chokeberry extract	Onopiuk et al., 2021 Poland [2]
		1 mg/kg Food 10 months				↑ TOS, ↑ OSI		
		5 mg/kg Food 10 months				↑ TOS, ↑ OSI, ↑ PC, ↓ CAT, ↓ GPx		
	Adult male Wistar rats	5 mg/dm^3^ in drinking water 12 months	SLG			↑ LPO, ↑ TOS/TAS	Zn	Kostecka-Sochoń et al., 2018 Poland [26]
		50 mg/dm^3^ in drinking water 12 months				↑ LPO, ↑ TOS, ↑ TOS/TAS		

ACAP: Antioxidant capacity against peroxyl radicals; AlCl_3_: aluminum chloride; CAT: catalase; Cd: cadmium; CK19: cytokeratin 19; DW: distilled water; Gpx: glutathione peroxidase; GSH: reduced glutathione; HE: hematoxylin eosin stain; HgCl_2_: mercury chloride; IHC: immunohistochemistry; IL-1β: interleukin-1β; LPO: lipid peroxidation; MDA: malondialdehyde; MeHg: methylmercury, MT I/II–anti-metallothionein I/II; OSI: oxidative stress index (=TOS/TAS), Pb: lead; PG: parotid gland; SLG: sublingual gland; SMG: submandibular gland; SOD: superoxide dismutase; TAS: total antioxidative status; TNF-α: tumor necrosis factor-alpha; TOS: total oxidative state; Zn: zinc; α-SMA: smooth muscle actin; ↓: decreased; ↑: increased. ^#^ only significant change vs. control: *p* < 0.05.

**Table 5:** Oxidative stress-induced histological and biomarkers changes induced by sodium fluoride on salivary gland experimental case-control studies.

Animals	Dose Route of Administration Duration	Salivary Gland	Histopathological Staining Technique	Histological Aspects	Oxidative Stress Markers ^#^	References
Pregnant Wistar rats and their offspring	10 mg sodium fluoride/L drinking water 42 days	PG SMG			↓ TEAC	dos Santos et al., 2022 Brazil [10]
	50 mg sodium fluoride/L drinking water 42 days	PG	HE α SMA, CK18	Increased ductal area	↑ TEAC	
		SMG		Increased ductal area	↓ TEAC	
Male Swiss Albino Mice	10 mg sodium fluoride/L drinking water 60 days	SMG			↑ GSH	Lima et al., 2018 Brazil [28]
	50 mg sodium fluoride/L drinking water 60 days	SMG			↑ GSH	
Male Wistar rats	15 mg sodium fluoride/kg Intraperitoneally a single injection	PG			↑ MDA, ↓ CAT	Yamaguti et al., 2013 Brazil [4]
		SMG			↑ MDA, ↑ CAT ↓ SOD after 1 h, ↑ SOD after 3 h, ↓ SOD after 24 h	

CAT: catalase; CK18: cytokeratin 18; GSH: reduced glutathione; HE: hematoxylin eosin stain; MDA: malondialdehyde; SOD: superoxide dismutase, TEAC: total antioxidant capacity; α-SMA: smooth muscle actin; ↓: decreased; ↑: increased. ^#^ only significant change vs. control: *p* < 0.05.

**Table 6:** Antioxidants with restorative effects on salivary glands’ oxidative stress changes.

Antioxidant	Dose Route of Administration Duration	Histopathological Staining Technique	Effects on Histological or Oxidative Biomarkers at the End of Treatment	Inducer of Oxidative Stress	References
Hesperidin	100 mg/kg/day Orally 7 days	HE Mallory α-SMA ki-67	Restauration of the normal level of MDA and GPx Anti-inflammatory effect	Cyclophosphamide	Mostafa et al., 2023 Egypt [15]
Selenium	0.2 mg/kg/day Orally 14 days	HE Bcl-2	Lack of apoptosis, lack of inflammation, and slight degenerative lesions	Cyclophosphamide	Alnuaimi et al., 2022 Iraq [12]
Febuxostat	10 mg/kg/day pre-treatment Orally 14 days	HE Toluidine-Blue α-SMA	Normal parotid histological structure, focal cell vacuolation, and moderate α-SMA immunoexpression	5-Florouracil	Abdelzaher et al., 2022 Egypt [16]
10-dehydrogingerdione from ginger extract	10 mg/kg/day Orally 45 days	HE PAS caspase-3	Normal architecture of cells and lack of apoptosis	Tramadol	Hassabou et al., 2021 Egypt [19]
Black chokeberry fruit	0.1% water solution of extract of polyphenols Orally 3 or 10 months		Significantly increased CAT after 3 months; CAT, SOD, and GPx after 10 months; and decreased TOS and OSI	Cadmium	Dąbrowska et al., 2019 Poland [27]
Black chokeberry extract	0.1% water solution black chokeberry extract daily Orally 3 or 10 months		Significant decrease in TOS and OSI after 10 months at both concentrations and after 3 months only for 5 mg of cadmium Significant increase in TAS and GPx after 3 and 10 month and SOD after 10 months	Cadmium	Onopiuk et al., 2021 Poland [2]
Zinc	Zn in different doses (30 mg Zn/L or 60 mgZn/L) Orally in drinking water 12 months		Significant increase in GSH after 5 mg Cd/L + 60 mg Zn/L and of TAS after 50 mg Cd/L + 60 mg Zn/L Significant decrease in TOS after 50 mg Cd/L + 60 mg Zn/L	Cadmium	Kostecka-Sochoń et al., 2018 Poland [26]
Glycyrrhizic acid	15 mg/kg/day Orally 3 months	HE MTC PAS CD68 α-SMA	Restored the levels of MDA, GSH, TAC, TNF-α, and IL-1β with no oxidative stress, no inflammation, and normal tissue architecture	Sodium nitrite	Elsherbini et al., 2020 Saudi Arabia, Egypt [31]

Bcl-2 (B cell lymphoma 2); CAT: catalase; Cd: cadmium; CD 68: cluster of differentiation 68 expressed by macrophages; Gpx: glutathione peroxidase; GSH: reduced glutathione; HE: hematoxylin eosin stain; ki67: a proliferation marker; IL-1β: interleukin-1β; MDA: malondialdehyde; MTC: Masson’s trichrome; PAS: periodic acid Schiff; OSI: oxidative stress index (=TOS/TAS), SOD: superoxide dismutase; TAS: total antioxidative status; TNF-α: tumor necrosis factor-alpha; TOS: total oxidative state; Zn: zinc; α-SMA: smooth muscle actin.

## Data Availability

Not applicable.

## Supplementary Materials

The following supporting information can be downloaded at: https://www.mdpi.com/article/10.3390/medicina59091692/s1.
