# Supplementary material for: Atrophy and Inflammatory Changes in Salivary Glands Induced by Oxidative Stress after Exposure to Drugs and Other Chemical Substances: A Systematic Review
Source: Medicina (Kaunas). 2023 Sep 21;59(9):1692. doi: 10.3390/medicina59091692 (PMC10535338; doi:10.3390/medicina59091692)
Supplement: Supplementary file 1 [file medicina-59-01692-s001.zip › medicina-2586864-supplementary.pdf]

Supplemental file

Table S2. Details regarding sample size, histopathology staining techniques used in experimental case-control studies on different strains of animals to show oxidative stress histological and biomarkers changes induced by drugs on salivary glands

**Autor Country Reference**	**Type of study Model considered**	**Sample size (number, drug)**	**Dose and route of administration**	**Sacrificed**	**How were anti-oxidants introduced**	**Histo-pathology staining technique**	**Observation and limitations**
Mostafa et al. 2003 Egypt [15]	Experimental case-control study Adult male albino rats	G1:10, control G2: 10, HSP G3: 10, CYP G4: 10, CYP+HSP	G1: control: normal saline for seven days G2: HSP (100 mg/kg body weight) daily orally for seven consecutive days G3: CYP (200 mg/kg body weight) single intraperitoneal injection on the 7th day of the experiment G4: HSP (100 mg/kg body weight) daily orally for seven consecutive days and CYP (200 mg/kg body weight) single intraperitoneal injection on the 7th day of the experiment	all animals were sacrificed; parotid glands were dissected and weighed. Then the tissues were distributed into two parts. The first one was homogenized and stored at − 80 °C for measuring of catalase activity, the levels of malondialdehyde (MDA) and glutathione peroxidase (GPx). The second part was prepared for histopathology and immunohistochemistry.	Pre-administration and coadministration	HE Mallory α-SMA ki-67	Salivary gland observation only during administration with no follow-up
Alnuaimi et al., 2002 Iraq [12]	Experimental case-control study Adult male albino rats	G1:6, control G2: 6, Se G3: 6, CYP G4: 6, CYP+Se	G1: control: normal slain orally using oral needle gavage for fourteen days G2: 0.2 mg /kg b.w. /day) of selenium orally that diluted in normal saline for fourteen days via oral needle gavage G3: 150 mg/kg of b.w.) of	At the end of the experiment (15th day), all rats in the four groups were anesthetized with an injection of ketamine and xylazine. Subsequently,	Pre-administration and coadministration	HE Bcl-2 Positive and negative controls were	No limitation identified

			cyclophosphamide (diluted in normal saline) injected intraperitoneally (i.p) on the 8th day.<br>G4: (0.2 mg /kg b.w. /day) of selenium orally for fourteen days, as well as, a single dose of (150mg/kg of b.w.) of cyclophosphamide diluted in normal saline were given on the day 8th of an experiment	submandibular salivary glands (SMG) were excised for histology and immunohistochemistry.		run simultaneously with biopsy specimen,	
**Abdelzaher et al., 2002**<br>Egypt<br>**[16]**	Experimental case-control study<br>Male albino rats	G1: 8, control<br>G2: 8, FEB<br>G3: 8 5-FU<br>G4: 8, FEB+ 5-FU	G1: 8 control group, was given only the vehicle for 14 days before receiving saline intravenous injections from the 10th to the 14th day.<br>G2: 8 FEB (10 mg/kg) [18] once daily po for 14 days, dissolved in 0.5% carboxymethylcellulose sodium (CMC)<br>G3: 8 5-FU (35 mg/kg/day) i.p from the 10th to 14th day<br>G4: 8 pre-treated with 10 mg/kg FEB orally for two weeks before receiving 5-FU administration from the 10th to the 14th day.	For histological and immunohistochemical tests, one gland from one side of each rat was excised	Pre-administration	HE<br>Toluidine-Blue<br>α-SMA	No limitation identified
Elhindawy et al., 2019<br>Egypt<br>[18]	Experimental case-control study<br>Adult male albino rats	G1:4, control<br>G2: 6, Tramadol<br>G3: 6, Tramadol withdrawal	G1: control 4 rats, received distilled water via gastric tube daily, one rat was sacrificed with each scarification period of other groups.<br>G2: Tramadol group.<br>6 rats, received 40 mg\kg 13 via gastric tube daily and were divided as follow:	20 days<br>30 days	Withdrawal<br>No antioxidant	H&E<br>Toluidine blue<br>capase-3	No limitation identified

			a) 3 rats received the drug for 20 days and then sacrificed.<br>b) 3 rats received the drug for 30 days and then sacrificed.<br>G3: Tramadol withdrawal group.<br>6 rats, received 40 mg\kg 13 via gastric tube daily for 30 days and were divided as follow:<br>a) 3 rats received distilled water for 20 days after tramadol withdrawal and then sacrificed.<br>b) 3 rats received distilled water for 30 days after tramadol withdrawal and then sacrificed.				
Onopiuk et al., 2018 Poland [5]	Experimental case-control study 14-month old male Wistar (Crl: WI (Han)) rats	G1: 8, control<br>G2:8, MTZ	G1: control:<br>Rats received drinking water intragastrically once a day in a volume of 0.5mL in the same time regimes as in the MTZ group. for 7 days<br>G2: MTZ: Rats received metronidazole intragastrically (tablets containing 250mg of active substance, Polpharma) once a day at a dose of 100 mg/kg body weight in a volume of 0.5mL of drinking water for 7 days	At the end of the experiment, 64 salivary glands, including 32 submandibular and 32 parotid (16 in each group), were collected from rats under anesthesia with ketamine (80 mg/kg) and xylazine (5 mg/kg).	NO antioxidant	No	The limitations during this project was the budget intended for the research. That conditioned the number of study groups and laboratory work. Furthermore, the main limitation of the study was limited publications in this subject

							that have similar animal model. Also, the research was executed on older rats. In addition, the study could be conducted only in an animal model. Therefore, this mechanism in the human body is not entirely clear and requires further research. In the future, more xenobiotics will be studied and its influence on oxidative stress.
Hassabou et al., 2021 Egypt	Experimental case-control	G1: 10, normal control	G1: normal control were fed plain chow, received no drugs	At the end of the experiment, all the rats were scarified then	coadministration	HE PAS of	The present study aimed mainly to

[19]	study Male albino rats	(NC) group. G2: 10, tramadol G 3: 10, 10-DHGD, G4: 10, tramadol + 10-DHGD	G2: tramadol intra peritoneal (20 mg /kg) body weight daily for 45 days. G3: received freshly extracted 10-DHGD, orally in a dose level (10 mg /kg) body weight and using 2% gum acacia as suspending agent for 45 days. G4: received tramadol and 10-DHGD in combination using the above-mentioned doses.	dissected, submandibular salivary glands (SMGs) tissues were removed instantly, rinsed with ice cooled normal saline and dried.		caspase-3	focus on histological alterations and apoptosis only of SMGs and dorsal tongue of experimental rats due to persistent tramadol intake. Despite the success demonstrated , there may be certain limitation in using extra parameters in concern to explain our proposal, however this will be taken in consideration during planning of further studies in the future.

*HSP: hesperidin, CYP: Cyclophosphamide, Se: selenium, FEB: febuxostat, 5-FU: 5-fluorouracil, MTZ: metronidazole, PAS: Periodic acid-Schiff

Table S3. Details regarding sample size, histopathology staining techniques used in experimental case-control studies on different strains of animals after ethanol exposure to show oxidative stress histological and biomarkers changes induced on salivary glands

**Autor Country Reference**	**Type of study Model considered**	**Sample size (number, drug)**	**Dose and route of administration**	**Sacri-ficed**	**How were anti oxidants introduced**	**Different groups considered or biopsied**	**Histo-pathology staining technique**	**Observation and limitations**
Ferreira et al., 2021 Brazil [20]	Experimental case-control study Rattus Novergicus, 60 days old,	G1: 4, control G2: 4, EtOH group	Pregnant dams were randomly divided into two groups (simple randomization), control and EtOH group (n = 4, each). Both groups were treated in gestational days GD6, GD7, GD8, with distilled water (control) or EtOH 3.0 g/kg (20 % w/vol) through intragastric gavage. After treatment periods, dams were kept under standard living conditions in single cages and have been weighted on GD13 and GD18. Two checkups after GD18 were performed daily for parturition verification. Day of birth was determined as postnatal day zero (PD0). Offspring groups were assigned at PD7. At PD20 males and females' pups were	-	No	Left side salivary glands (Parotid gland and Submandibular gland) were assigned for some analyses (Lipid peroxidation, Nox metabolites evaluation by Griess reagent assay, and Antioxidant Capacity against Peroxils Radicals – ACAP). Right side salivary glands (PG and SMG)	HE CK-19 vimentin	The objective of pilocarpine was to stimulate salivary secretion in both groups at same condition. Pilocarpine is commonly used for cases of xerostomia due to its effects on muscarinic M3 receptors and it was used due to spontaneous saliva collection limitations regarding amount of collected

			weaned separated in different cages respecting treatment groups and weighted weekly until PD40.					saliva [20].
Fagundes et al., 2016 Brazil [7}	Experimental case-control study Female Wistar rats, 35 days old	G1: 5, control G2: 5, control G3: 8, ethanol G4: 8, ethanol	G1, G2 – control groups distilled water G3, G4: one group, with 1 week of 3 days of exposure to ethanol/distilled water; and another group, with 4 weeks of 3 days of exposure to ethanol/distilled water. Each group was composed of a control group, where the animals received distilled water, and an ethanol group, where animals received EtOH. Twelve hours after treatment, animals were divided and submitted to collection of fresh glands or perfusion (*n* = 5–8 animals per group,	-	No antioxidant	The gland of the right side was removed and used for oxidative stress analysis. The gland of the left side was removed after perfusion and used to immunohistochemistry and morphometric analysis. All glands were weighe	α-SMA, CK-18 VIM	No limitation identified
Fernandes et al., 2015 Brazil [6]	Experimental case-control study adolescent female Wistar rats, 35 days old	G1: 8, control G2: 8, ethanol	G1: control: distilled water (n=8) G2: ethanol (6.5 g/kg/day, 22.5% w/v) (n=8). Every day, rats were administered through orogastric	-	No antioxidant	After 7.5 hours of the last administration of alcohol, which is the period related to non-detectable amounts of ethanol in the blood (Livy et al., 2003),	α-SMA, CK19 CAS	No limitation identified

			cannula over a period of 55 days (i.e., until the 90th day of life)			animals were anaesthetised with a mixture of ketamine hydrochloride (72 mg/kg, i.p.) and xylazine (9 mg/kg, i.p.) and then perfused with heparinized saline followed by paraformaldehyde 4%. The PG and SG were removed and weighed by analytical balance After weighing, glands were post-fixed in formalin solution (10%), and submitted to histological process by dehydration in increasing alcohol battery and xylene, and finally embedded in Paraplast® resin.		
Sorkina et al., 2022 Russia [9]	Experimental case-control study mature male albino rats of the Wistar line	G1: 60, control G2: 20, ethyl alcohol 30 days G3: 20, ethyl alcohol 120 days	G1 was the control group. The experimental groups of animals was injected with 6.9 g/kg/day of 20% ethyl alcohol solution through the oral cavity with a syringe. G2 was treated for 30	-	No atioxidant	Then, large SMG and PG were collected and subjected to morphological and histochemical research methods. The body weight of the rats and salivary glands was measured at each	HE toluidine blue	No limitation identified

		G4: 20, ethyl alcohol 180 days	days, G3 was treated for 120 days (n ¼ 20), G4 was treated for 180 days.			experimental period.		

* α-SMA: Anti-α muscle actin, CK-18: anticytokeratin-18, VIM: vimentin, CK19: cytokeratin 19, CAS:

Table S4. Details regarding sample size, histopathology staining techniques used in experimental case-control studies on different strains of animals to show oxidative stress histological and biomarkers changes induced by heavy metals on salivary glands

**Autor Country Reference**	**Type of study Model considered**	**Sample size (number, drug)**	**If any controls were considered**	**Dose and route of administration**	**Sacrificed**	**How were anti oxidants introduced**	**Different groups considered or biopsied**	**Histopathology staining technique**	**Observation and limitations**
Souza-Monteiro et al., 2020 Brazil [11]	Experimental case-control Swiss albino mice	G1: 10, control G2: 10, $AlCl_3$	G1: control group	G2: Al chloride ($AlCl_3$) (18.5 mg/kg) with distilled water as vehicle by intragastric gavage for 60 days.	-	No antioxidant	The salivary glands were removed and conducted to histological procedures. For this analysis, we evaluated the area of the tissues and their structures.	HE	No limitation identified
Souza-Monteiro et al., 2022 Brazil [21]	Experimental case-control Male Wistar rats	G1: 16, control G2: 16, aluminum	The control group received only distilled water (H_2Od) following the same protocol.	The aluminum group received 8.3 mg/kg/day of $AlCl_3$ diluted in 8 mg/mL distilled water daily for 60 days. The exposure solution was administere	-	No antioxidant	the salivary glands were collected and then stored at -80 ^{0}C for further biochemistry and proteomic assays. The remaining eight animals per group were perfused for histological analysis.	HE	No limitation identified

				d by intragastric gavage.					
Aragão et al., 2017 Brazil [22]	Experimental case-control Male albino rats	G1:20, control G2: 20, mercury chloride (HgCl2)	In the control group (n = 20), only distilled water was administered in similar volumes. All animals were submitted to oral intragastric gavage for 45 days.	The intoxicated group (n = 20) received an oral dose of 0.375 mg/kg/day of mercury chloride (HgCl2), according to the protocol previously described by Szasz et al.13 and Teixeira et al.14., 45 days	yes	No antioxidant	After the intoxication period, 10 animals from each group were perfused, with the collection of submandibular and parotid glands, followed by salivary glands weight measurement on analytical balance (FA 2104N, Bioprecisa Electronic Balance, Shanghai, China). After this, these glands were sent to tissue processing and subsequent morphometric and immunohistochemical analysis. The non-	anti-MT - I/II anti-Cytokeratin 19 (CK-19)	No

							perfused animals were sacrificed, with collection of submandibular and parotid gland. These glands were submitted to cold-vapour atomic absorption (CVAA) spectroscopy		
Farias-Junior et al., 2017 Brazil [25]	Experimental case-control Male Wistar rats, 40 days of age	G1: 20, control G2: 20, methylmercury (diluted in corn oil)	the other group received only corn oil, 35 days	One group received methylmercury (diluted in corn oil) at a dose of 0.04 mg/kg/day 35 days	After 35 days of intoxication, the animals were submitted to cervical dislocation, with the dissection of parotid and submandibular salivary glands.	No antioxidant	-	No histology	No limitation identified
Lopes et al., 2020 Brazil [23]	Experimental case-control Male Wistar	G1: 20, control G2: 20, Pb acetate	Animals in the control group (n = 20) received similar	Pb acetate (Sigma-Aldrich, Germany) was used	After 55 days of exposure, 10 animals from each	No antioxidant	The remaining 10 animals from each group were perfused, and their parotid	HE MT I/II α-SMA	No limitation identified

	rats		volume of distilled water, 55 days	and administered to the animals of the exposed group (n = 20) by intragastric gavage at a concentration of 50 mg/kg/day, 55 days	group were euthanized, and their blood and salivary glands were collected. The salivary glands were washed in saline solution, frozen in liquid nitrogen, and stored at − 80 °C. Then, the samples were sonically homogenized in Tris-HCl buffer (20 mM, pH 7.4) until the approximate concentration of 1 g/mL		and submandibular glands were collected for histological procedures for morphometric and immunohistochemical analyses.		

					and proceed the biochemical analyses.				
Dąbrowska et al., 2019 Poland [27]	Experimental case-control 96 young (3-4-weekold) female Wistar	G1: 16, control G2: 16, AMPF G3: 16, Cd1 G4: 16, Cd1 + AMPF G5: 16, Cd5 G6: 16, Cd5 + AMPF	Group 1—control: throughout the experiment (3 or 10 months), the rats received purified water and standard fodder (Labofeed, Kcynia)	Group 2—AMPF: the rats received only a 0.1% water solution of extract of polyphenols (AMPF) for drinking for 3 or 10 months and standard fodder Group 3—Cd1: the rats were exposed to cadmium (as $CdCl_2$) in the fodder, receiving 1mg Cd/kg for 3 or 10 months, and received purified	-	APMF Co-administration	-	No histology	No limitation identified

				water for drinking<br>Group 4—Cd1 + AMPF: throughout the period (3<br>or 10 months) of exposure to 1mg of Cd/kg, the rats received the 0.1% water solution of extract of polyphenol s<br>from black chokeberry fruit<br>Group 5—Cd5: the rats received fodder containing 5mg<br>Cd/kg for 3 or 10 months and purified water for					

				drinking Group 6—Cd5 + AMPF: during exposure (for 3 or 10 months) to 5mg Cd/kg of fodder, the rats received 0.1% water solution of extract of polyphenols from black chokeberry fruit for drinking					
Onopiuk et al., 2021 Poland [2]	Experimental case-control 96 female Wistar rats	G1: 16, control G2 G3 G4 G5 G6	-	(control group and ChE group) or containing cadmium (Cd1 group, Cd5 group, Cd1 +ChE group, and Cd5+ChE group) and drinking	After 3 and 10 months of the experiment, the right and left sublingual salivary glands were collected under general	ChE coadministration	-	No histology	The next limitation is the small weight of the sublingual salivary glands, which made it impossible to perform a wider range of studies, including determination

				fluid—unpolluted water (control group, Cd1 group, and Cd5 group) or 0.1% aqueous ChE (ChE group, Cd1+ChE group, and Cd5+ChE group). The 0.1% ChE was prepared from the powdered A. melanocarpa extract	anaesthesia with barbiturate (intraperitoneal administration of Morbital in a dose of 30 mg/kg b.w.), immediately after animals were sacrificed. For this purpose, the common sublingual and submandibular connective tissue capsule, covering both salivary glands, was removed, and the right and left sublingual				of indices of oxidative modifications of nucleic acids and evaluation of the microscopic structure of the salivary gland tissue. Another limitations of the study are the impossibility of explanation of the molecular pathways of the preventive action of ChE with respect to the oxidative stress and oxidative modifications of proteins and lipids induced by cadmium in the sublingual salivary gland, as well as whether the

					glands (the sublingual glands are paired salivary glands of the same structure and function) were separated from the submandibular glands.				changes in the balance between the processes of oxidation and reduction in this salivary gland noted due to the administration of cadmium or/ and ChE might be the outcome or the cause of the impact on the salivary glands' secretory function or saliva composition. In the available literature, we have found no data on the relationship between salivary glands' oxidative/reductive status and the saliva secreted by

									them.
Kostecka-Sochoń et al., 2018 Poland [26]	Experimental case-control 72 adult male Wistar rats	9 groups, and in each group was 8 animals. In research, 2 groups received Zn alone, 2 groups were treated with Cd alone, and 4 groups received Zn supplementation during exposure to Cd.	The control group received drinking water without cadmium or zinc.	Zn and Cd were administered in drinking water at the concentrations of 30 or 60mg Zn/L (as ZnCl2; Merck) and 5 or 50mg Cd/L (as CdCl2·2 1/2H2O; POCH; Gliwice, Poland) alone (30mg Zn/ L, 60mg Zn/L, 5mg Cd/L, and 50mg Cd/L groups) and in combination (5mg Cd/L + 30mg Zn/L, 5mg Cd/L + 60mg Zn/L, 50mg Cd/L +	-	Zn coadministration	-	No histology	No limitations identified

				30mg Zn/L, and 50mg Cd/L + 60mg Zn/L groups) for up to 12 months.					

* AMPF: water solution of extract of polyphenols; ChE: chokeberry extract

Table S5. Details regarding sample size, histopathology staining techniques used in experimental case-control studies on different strains of animals to show oxidative stress histological and biomarkers changes induced by sodium fluoride on salivary glands

**Autor Country Reference**	**Type of study Model considered**	**Sample size (number, drug)**	**If any controls were considered**	**Dose and route of administration**	**Sacrificed**	**How were anti oxidants introduced**	**Different groups considered or biopsied**	**Histopathology staining technique**	**Observation and limitations**
dos Santos et al., 2022 Brazil [10]	Experimental case-control Pregnant Wistar rats (Rattus norvegicus) aged 90 days (150–200 g).	-	-	Nine pregnant Wistar rats (n = 3/group) were randomly distributed into three experimental groups: 0 mg F/L (control group—deionized water), 10 mg F/L, and 50 mg F/L groups (after sodium fluoride solubilization in ultrapure water). All groups received water with their receptive concentratio	-	No antioxidant	For this, after perfusion, the salivary glands were removed and post-fixed in 4% formaldehyde until processing.	HE α-SMA, CK-18	Although the present study brings unprecedented data on the effects of indirect exposure to F on salivary glands, an important limitation deserves comment, as the period that the salivary glands were analyzed, (i.e., 21 days post-natal) might not have allowed a complete maturation of the glands.

				ns of F and food under ad libitum conditions. Each pregnant rat generated approximately 10 offspring, comprising 30 offspring. Then, randomization was made to define 10 offspring per group. Only male animals were used in this study, and the remaining animals were used for another study. The experimental groups were conditioned to the exposure protocol with					

				their respective concentrations for 42 days, out of which the first 21 days comprised the gestation period of the rats, and the following 21 days, the lactation period of the offspring. The doses used in this study 10 mg F/L and 50 mg F/L represent, respectively, 1–2 mg F/L and 5–10 mg F/L consumed by humans;					
Lima et al., 2018 Brazil [28]	Experimental case-control Control group (n=20)were fed	Ninety-day-old male Wistar rats (240 g approximately; n = 40)	Animals in the MeHg group were fed with 0.04 mg/kg/day of MeHg in oil corn (n = 20) by intragastric	-	No antioxidant	Ten animals of each group were used to tissue	The parotid, submandibular, and sublingual glands were removed, post	HE α-SMA, CK-18 MT I/II	No limitations identified

	onlywith oil corn respecting the v/v ratio.		gavage over 60 days			analysis.	fixed in 4% formaldehyde until processing.		
Yamaguti et al., 2013 Brazil [4]	Experimental case-control -	One hundred two-month-old male rats of Wistar strain were used in the present investigation.	Animals weighed between 220–270 g and, therefore, were randomly and equally (*n* = 50) stratified into two groups according to the treatment received, fluoride (F) and control (C). Fluoride treatment groups were intraperitoneally administered with a single injection of NaF solution (15 mg F−/kg b.w.), and control rats received an equivalent dose of sodium chloride solution (0.9% NaCl). Each	-	No antioxidant	-	-	No histology	No limitations identified

			treatment group, F and C, was further divided into 5 subgroups according to the length of time after injection. The animals were euthanized 1, 3, 6, 12, and 24 h after injection, and SM and PA salivary glands were immediately excised, cleaned in isotonic solution, precooled in dry ice, and stored at −80∘C until further processing.						

*MT I/II - anti-metallothionein I/II

Table S6. Details regarding sample size, histopathology staining techniques used in experimental case-control studies on different strains of animals to show oxidative stress histological and biomarkers changes induced by antioxidants with restorative effects on salivary glands

**Autor Country Reference**	**Type of study Model considered**	**Sample size (number, drug)**	**If any controls were considered**	**Dose and route of administration**	**Sacrificed**	**How were anti oxidants introduced**	**Different groups considered or biopsied**	**Histopathology staining technique**	**Observation and limitations**
Mostafa et al., 2023 Egypt [15]	Experimental case-control study Adult male albino rats	G1:10, control<br>G2: 10, HSP<br>G3: 10, CYP<br>G4: 10, CYP+HSP	G1: control: normal saline for seven days	G1: HSP (100 mg/kg body weight) daily orally for seven consecutive days<br>G2: CYP (200 mg/kg body weight) single intraperitoneal injection on the 7th day of the experiment<br>G4: HSP (100 mg/kg body weight) daily orally for seven consecutive days and CYP (200 mg/kg body weight) single intraperitoneal injection on the 7th day of the experiment	all animals were sacrificed; parotid glands were dissected and weighed. Then the tissues were distributed into two parts. The first one was homogenized and stored at − 80 °C for measuring of catalase activity, the levels of malondialdehyde (MDA) and glutathione peroxidase (GPx). The second part was prepared for histopathology and immunohistoche	Pre-administration and coadministration	G1<br>G2<br>G3<br>G4	HE<br>Mallory<br>α-SMA<br>ki-67	Salivary gland observation only during administration with no follow-up

					mistry.				
Alnuaimi et al., 2022 Iraq [12]	Experimental case-control study Adult male albino rats	G1: 6, control G2: 6, Se G3: 6, CYP G4: 6, CYP+Se	C1: control: normal slain orally using oral needle gavage for fourteen days	G2: 0.2 mg /kg b.w. /day) of selenium orally that diluted in normal saline for fourteen days via oral needle gavage G3: 150 mg/kg of b.w.) of cyclophosphamide (diluted in normal saline) injected intraperitoneally (i.p) on the 8th day. G4: (0.2 mg /kg b.w. /day) of selenium orally for fourteen days, as well as, a single dose of (150mg/kg of b.w.) of cyclophosphamide diluted in normal saline were given on the day 8th of an experiment	At the end of the experiment, all rats in the four groups were anesthetized with an injection of ketamine and xylazine. Subsequently, submandibular salivary glands (SMG) were excised for histology and immunohistochemistry.	Preadministration and coadministration	G1 G2 G3 G4	HE Bcl-2 Positive and negative controls were run simultaneously with biopsy specimen,	No limitation identified
**Abdelzaher** et al., 2022 Egypt	Experimental case-control study	G1: 8, control G2: 8, FEB G3: 8, 5-FU	G1: 8 control group, was	G2: 8 FEB (10 mg/kg) [18] once daily po for 14 days, dissolved	For histological and immunohistochemical tests, one	Pre-administration	G1 G2 G3 G4	HE Toluidine-Blue α-SMA	No limitation identified

[16]	Male albino rats	G4: 8, FEB+ 5-FU	given only the vehicle for 14 days before receivin g saline intraven ous injection s from the 10th to the 14th day.	in 0.5% carboxymethylce llulose sodium (CMC) G3: 8 5-FU (35 mg/kg/day) i.p from the 10th to 14th day G4: 8 pre-treated with 10 mg/kg FEB orally for two weeks before receiving 5-FU administration from the 10th to the 14th day.	gland from one side of each rat was excised				
Hassabo u et al., 2021 Egypt [19]	Experime ntal case-control study Male albino rats	G1: 10, normal control (NC) G2: 10, tramadol G 3: 10, 10-DHGD, G4: 10, tramadol + 10-DHGD	G1: normal control were fed plain chow, received no drugs and served as	G2: tramadol intra peritoneal (20 mg /kg) body weight daily for 45 days. G3: received freshly extracted 10-DHGD, orally in a dose level (10 mg /kg) body weight and using 2% gum acacia as suspending agent for 45 days. G4: received tramadol and 10-DHGD in	At the end of the experiment, all the rats were scarified then dissected, submandibular salivary glands (SMGs) tissues were removed instantly, rinsed with ice cooled normal saline and dried.	coadministrat ion	G1 G2 G3 G4	HE PAS of caspase-3	The present study aimed mainly to focus on histological alterations and apoptosis only of SMGs and dorsal tongue of experimental rats due to persistent tramadol intake. Despite the success demonstrated

				combination using the above mentioned doses.					, there may be certain limitation in using extra parameters in concern to explain our proposal, however this will be taken in consideration during planning of further studies in the future.
Dąbrowska et al., 2019 Poland [27]	Experimental case-control 96 young (3-4-weekold) female Wistar	G1: 16, control	G1: control, throughout the experiment (3 or 10 months), the rats received purified water and standard fodder (Labofeed, Kcynia)	Group 2—AMPF: the rats received only a 0.1% water solution of extract of polyphenols (AMPF) for drinking for 3 or 10 months and standard fodder Group 3—Cd1: the rats were exposed to cadmium (as $CdCl_2$) in the fodder, receiving 1mg Cd/kg for 3	-	APMF Co-administration	-	No histology	No limitation identified

				or<br>10 months, and received purified water for drinking<br>Group 4—Cd1 + AMPF: throughout the period (3<br>or 10 months) of exposure to 1mg of Cd/kg, the rats received the 0.1% water solution of extract of polyphenols from black chokeberry fruit<br>Group 5—Cd5: the rats received fodder containing 5mg Cd/kg for 3 or 10 months and purified water for drinking<br>Group 6—Cd5 + AMPF: during exposure (for 3 or 10 months) to 5mg Cd/kg of fodder, the rats received<br>0.1% water solution of extract of					

				polyphenols from black chokeberry fruit for drinking					
Onopiuk et al., 2021 Poland [2]	Experimental case-control 96 female Wistar rats	G1: 16, control G2 G3 G4 G5 G6	-	(control group and ChE group) or containing cadmium (Cd1 group, Cd5 group, Cd1 +ChE group, and Cd5+ChE group) and drinking fluid—unpolluted water (control group, Cd1 group, and Cd5 group) or 0.1% aqueous ChE (ChE group, Cd1+ChE group, and Cd5+ChE group). The 0.1% ChE was prepared from the powdered A. metacarpal extract	After 3 and 10 months of the experiment, the right and left sublingual salivary glands were collected under general anesthesia with barbiturate (intraperitoneal administration of Morbital in a dose of 30 mg/kg b.w.), immediately after animals were sacrificed. For this purpose, the common sublingual and submandibular connective tissue capsule, covering both salivary glands, was removed, and the right and left sublingual glands (the sublingual	ChE coadministration	-	No histology	The next limitation is the small weight of the sublingual salivary glands, which made it impossible to perform a wider range of studies, including determination of indices of oxidative modifications of nucleic acids and evaluation of the microscopic structure of the salivary gland tissue. Another limitations of the study are the impossibility of explanation of the

					glands are paired salivary glands of the same structure and function) were separated from the submandibular glands.				molecular pathways of the preventive action of ChE with respect to the oxidative stress and oxidative modifications of proteins and lipids induced by cadmium in the sublingual salivary gland, as well as whether the changes in the balance between the processes of oxidation and reduction in this salivary gland noted due to the administratio n of cadmium or/ and ChE might be the outcome or the cause of the impact on

									the salivary glands' secretory function or saliva composition. In the available literature, we have found no data on the relationship between salivary glands' oxidative/reductive status and the saliva secreted by them.
Kostecka-Sochoń et al., 2018 Poland [26]	Experimental case-control 72 adult male Wistar rats	9 groups, and in each group was 8 animals. In research, 2 groups received Zn alone, 2 groups were treated with Cd alone, and 4 groups received Zn supplement	The control groups received drinking water without cadmium or zinc.	Zn and Cd were administered in drinking water at the concentrations of 30 or 60mg Zn/L (as $ZnCl_2$; Merck) and 5 or 50mg Cd/L (as $CdCl_2 \cdot 2\ 1/2H_2O$; POCH; Gliwice, Poland) alone (30mg Zn/ L, 60mg Zn/L, 5mg Cd/L, and	-	Zn coadministration	-	No histology	No limitations identified

		ation during exposure to Cd.		50mg Cd/L groups) and in combination (5mg Cd/L + 30mg Zn/L, 5mg Cd/L + 60mg Zn/L, 50mg Cd/L + 30mg Zn/L, and 50mg Cd/L + 60mg Zn/L groups) for up to 12 months.					
Elsherbini et al., 2020 Saudi Arabia, Egypt [31]	Experimental case-control -	Thirty male Sprague Dawley rats weighing 100–150 g	-	3 groups. (1) Control group received phosphate buffer solution. (2) Sodium nitrite-treated (SN) group, rats were treated with (80 mg/kg) SN dissolved in distilled water (Sigma Aldrich, St Louis, MO, USA) (3, 8). (3) Glycyrrhizic acid (GA) treated group, rats were given 15 mg/kg GA, dissolved in distilled water (Sigma-Aldrich, St Louis, MO, USA)	All rats were anesthetized then euthanized by overdose of halothane.20 Both SMG and lung specimens were collected and processed for further analysis. The sublingual gland was separated from submandibular, sublingual gland complex before processing.	Glycyrrhizic acid (GA) treated group pretreatment	-	The right halves of specimens were fixed, processed into 5 mm paraffin sections, and stained with Hematoxylin and Eosin (H&E),21 Masson's Trichrome (MTC) stain to detect collagen fibers, and Periodic Acid Schiff (PAS)	No limitation identified

				followed by SN (80 mg/kg). All treatment modalities were given orally and daily for 3 months.				CD68 α-SMA	
